# Case of Axillary Aneurism Successfully Treated by Tying the Subclavian Artery

**Published:** 1831

**Authors:** 


### CASE OF AXILLARY ANEURISM SUCCESSFULLY TREATED BY TYING THE SUBCLAVIAN ARTERY. By CHARLES MAYO, Esq. Surgeon to the County Hospital in Winchester.

[Med. Chir. Trans.]

TEN years ago Mr. Mayo gave some account of a similar disease unsuccessfully treated by the same means. It is consolatory that he is now able to present to the public a more fortunate issue.

*Case.* On the 19th March, 1831, W. Matthews, aged 49, applied to Mr. M. respecting a painful affection of the right breast, of about a month's duration. On examination, a tumour was seen beneath the left clavicle, and soon ascertained to be aneurismal, and of the subclavian artery, at the point where it assumes the name of axillary. He was bled to 20 ounces, and took antimony and digitalis. No particular relief was obtained, and, on the 24th of the same month, he was taken into the Winchester Hospital. A consultation decided on the necessity of an operation, and the following is the account of its execution.

"March 26th. At ten this morning, with the assistance of my colleagues, I proceeded to the operation, and drawing down the skin of the neck, I made an incision about three inches and a half in length on the surface of the left clavicle, extending from the insertion of the sterno-cleido mastoideus muscle to the clavicular portion of the trapezius; by this the platysma myoides was exposed, which as well as the subjacent fascia I carefully divided, for upon the latter many branches of the external jugular vein were found, several of which I was obliged to divide in the progress of my dissection through the cellular substance, and secure them with ligatures. I traced the edge of the omohyoideus muscle, traversing the upper part of the wound, and directly below it I could place my finger on the artery as it passed over the first rib, which seemed to be about an inch and a half or two inches from the surface; to this point I directed all my attention, and endeavoured to clear my way to the artery by cautious touches with the edge of the scalpel, and by tearing the cellular substance with its handle and with a director, till at length I was able to get my nail upon the rib and then under the artery, so that after various efforts I passed a common blunt aneurismal needle under it, armed with a strong round ligature, and having ascertained that nothing else but the artery was included in the ligature, I tied it with a double knot, drawing each knot tight with the iron rings invented by the late Mr. Ramsden. The subclavian vein appeared just within and below the superior border of the clavicle, but formed no impediment to the operation; the branches of the external jugular, however, were very annoying, and kept the wound continually filled with blood, and the apprehension of wounding larger branches limited the extent of the internal wound to two inches at most. He bore the operation with great courage, though with some impatience, as it occupied rather more than twenty minutes; the pulsation ceased, and the pains in the shoulder were much relieved. In the evening he felt his neck very stiff and sore, and was harrassed with a short cough, which shook him, without enabling him to expectorate. He took tea and gruel, and had an opiate at bed time.

27th. Had passed a tolerable night, but was sick and faint this morning, which may be attributed to the opiate; took Pil. Cambog. C. gr. v. which moved his bowels two or three times. Pulse eighty-four; left hand and arm quite warm, but feeling heavy, and no pulse at the wrist; wound easy, tumour greatly subsided. Being feverish and worried with cough at night, I took f℥viij. of blood from the arm, which was slightly buffed."

We shall pass over the diurnal details. We find the patient, on the 10th of May, discharged cured. The wound was completely cicatrized—the tumour had disappeared, and the arm was recovering strength. The pulse was not perceptible at the wrist.

We congratulate Mr. Mayo on the result of a difficult operation and a dangerous disease.

